# Be Thankful

**Published:** 1859-03

**Authors:** 


### BE THANKFUL.

VERY many persons fail to enjoy what they have in the eating anxiety for what they have not, forgetting all the while how much they are in advance of others quite as good as they are, but whose days are blackness, whose every breath is in pain, and who feed on tears and sighs.

Valentine Perkins, of Mantua, Ohio, aged forty-five years, has every joint in his body as immovable as a solid bone, except those of two toes and two fingers. His jaws have been set and motionless for thirty years, the only aperture through which he receives food being that made by the falling out of his front teeth. His appetite is uniformly good. There was in London an old man who was called the Judge; he was always the picture of neatness, cheerfulness, and content. His wife, poor soul, is all but bed-ridden; he can only do half a day's work, and kind friends make up the remainder.

A correspondent writes from Virginia:

CARRSVILLE, VA., August 27th, 1858.

DEAR DR. HALL:

I was most pleasantly surprised a few days since by a very unexpected visit from a once familiar friend and guest—your highly and deservedly popular little Journal. From the chirography of the superscription upon the envelope, which I recognized in a moment, as I would the features of an old acquaintance, I suppose I am indebted to you for the favor; and it is to thank you, most warmly thank you for the rich intellectual repast which it afforded me, that I venture to intrude upon you. Be sure it found no ungrateful recipient or unappreciative reader.

Last year, through the kindness of a considerate friend, it was placed upon my pillow on each successive month. Since then, adverse circumstances have forced me to forego the indulgence; but long years of suffering and dependance have taught me the lesson of self-abnegation. And now a word as to my health, of which you may possibly entertain some curiosity to be informed. Six years from the incipient attack—four and a half from my prostration upon my couch—find me yet struggling with a relentless monster; yet as rigid and as helpless as a mass of stone, my eyes and tongue being the only members over which I have the least control. Recently my constantly increasing debility and emaciation admonish me that I shall soon receive a summons from beyond the dark valley. An almost unmanageable constipation of the bowels, together with a distressing asthmatic affection, are steadily but surely wasting away my constitution, which, until some time past, resisted disease with astonishing obstinacy. My digestive organs have lost so much of their energy, and have grown so torpid, that it is extremely difficult for me to effect an operation upon my bowels. An interim of two or three weeks frequently occurs between evacuations. The inability to open my jaws forces me to subsist upon such food as I can compress through a cavity made by the loss of two of my teeth, such as baked fruit, milk, boiled custards, half-cooked eggs, toasted bread, &c., &c.

W. H. E.

Not many will read these statements without a deep sympathy for these afflicted men, and an earnest hope that as to each one of them the sufferings of this life will bear no proportion to the high happiness in reserve for them in the heavenly world—a happiness as pure as a sunbeam—as eternal as the throne of God. On the other hand, let us all turn our attention in upon ourselves and cultivate a deep and an abiding gratitude to the Giver of all good, in that we and ours have been born perfect in limb, and form, and feature; our bodies without a blemish, our minds without a blot, and, further, that these things have been continued to us for the period of a life time, and that we have had given to us all things richly to enjoy by a Beneficence as ceaseless as the flow of time, and as boundless as the universe.

